# A Preliminary Set of Principles to Support Learning in the Context of Generative AI

**DOI:** 10.1021/acs.jchemed.5c01413

**Published:** 2025-12-26

**Authors:** Melanie M. Cooper

**Affiliations:** Chemistry Department, 3078 Michigan State University, East Lansing, Michigan 48224, United States

**Keywords:** First-Year Undergraduate/General, Curriculum, Generative AI, Learning Theories, Student-Centered Learning

## Abstract

Generative AI chatbots are now widely available, and increasing numbers of students use them. Despite hyperbolic claims, there is little objective evidence of the efficacy of such systems for teaching and learning. Early findings suggest that the use of AI chatbots without guidance or guardrails negatively affects student learning. Using what evidence we do have together with our current understanding of how people learn, I lay out a set of tentative principles for using generative AI to support learning and instruction Given the overarching assumption that *learning requires effort and engagement which can easily be bypassed using AI*, I propose four practical principles to guide faculty as they maneuver through this new era. (1) *Design AI teaching and learning systems to support self-regulated learning*. (2) *Develop a course structure and culture that rewards the learning journey*. (3) *Take advantages of the affordances of AI to extend what students know and can do*. And (4) *develop clear and equitable policies for the use of AI*. These principles are predicated on the idea that typical traditional courses (where emphasizing facts and algorithmic problem solving are emphasized) will become obsolete as these tasks are easily (and perhaps better) carried out by AI bots. It will become increasingly important for students to understand how they learn, what they can do with their knowledge, and how to solve complex problems that have societal and economic value.

## Graphical abstract

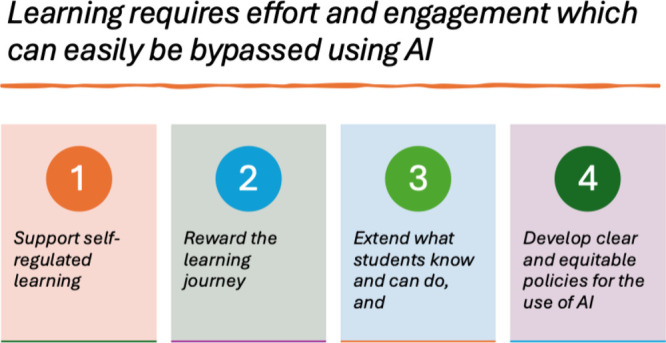

## Introduction

Generative AI burst into public consciousness in late 2022, and since then, its capabilities and ubiquity have advanced rapidly. Over the past two years, AI has been embedded into many of the systems we encounter daily. As of June 2025, ChatGPT had over 800 million daily users,^1^ and it is estimated that “Moore’s Law” (the doubling time for capability) for GenAI is seven months.^2^ The use of GenAI for education has been enthusiastically but asymmetrically adopted, by which I mean the people who are on the receiving end of education compared to those who aim to deliver that education. While estimates vary (since these data are self-reports) it appears that over 85% of students have used GenAI to complete assignments as part of their formal education.^3^ There has been no parallel burst of enthusiasm from most higher education institutions or instructors. A survey of faculty indicated that 80% of faculty felt unprepared to use AI.^4^

Certainly, there are numerous publications on the use of GenAI in education authored by early adopters^5^ (over 5000 and counting), but many educators are only just beginning to grapple with the impact and implications of the GenAI revolution. There are several reasons for this, including the fact that, for many faculty, the first use of AI they have encountered is inappropriate student use on assignments not designed for AI, and the tendency of systems to hallucinate.

Almost three years into the AI era, most higher education institutions have failed to provide instructors with the tools needed to investigate the affordances and drawbacks of this rapid change. In fact, we do not really know how to use GenAI to support student learning.^4^ This lack of evidence has not stopped numerous commercial entities from making unfounded claims about how their AI systems improve student learning and make instructors lives easier by removing “tedious” (or vital and important, depending on how you look at it) tasks such as designing instruction and instructional materials and grading student work. In 2024, there were over 1500 start-up AI learning systems, funded by over $6.5 billion,^6^ to say nothing of the commercial entities already in the field who are embedding AI into their existing systems. Similarly, the major AI companies are investing heavily in AI for education and providing free access to studens^7,8^ to secure a future user-base. The current U.S. Presidential administration of Donald J. Trump has released an official proclamation^9^ calling for increased use of AI in education.

The claims about the affordances of these commercial systems are unfounded because we do not have a great deal of careful research on outcomes (that is, their effects on students). Such research takes time, resources, and dare I say it–expertise. I think we can be fairly confident in survey studies that tell us students “like” GenAI supported learning systems and find them useful.^3^ After all, chatbots are specifically designed to be helpful and accommodating (or depending on how you look at it ingratiating and obsequious); they can provide individual tutoring, support, and feedback, and they can take the hard work out of completing assignments (and for those so inclined, can actually do all the work), so what’s not to like? However, there is no robust body of work on the impact of GenAI on actual learning (rather than on the perception of learning). As the New York Times wrote, “*OpenAI’s push to A.I.-ify college education amounts to a national experiment on millions of students. The use of these chatbots in schools is so new that their potential long-term educational benefits, and possible side effects, are not yet established*.”.^10^

In fact, the research we do have is not particularly encouraging. While most of the research on the impact of AI on learning leans on participant self-reports, few measure learning in any meaningful way. Those reports that do investigate aspects of cognition are not so positive. For example there are a number of studies that appear to show that use of AI tends to reduce critical thinking, cognitive effort,^11–13^ and exam performance.^14,15^ When students use AI for homework, students found difficulty with the summative tests when AI was unavailable.^15^ This finding correlates with an earlier study^16^ predating the use of AI, showing the number of students who benefited from doing homework decreased from 2008 to 2017. This study timeline corresponds to the rise of widely available Internet search systems and online “homework assistance” companies such as Chegg^17^ or Course Hero.^18^ The rise of much more powerful and accessible AI systems will exacerbate this issue unless we change our approaches to teaching and learning and how we gather evidence about what students know and can do.

So, while we do not currently have a huge amount of evidence about the impact of AI on learning, we can make some reasonable inferences: (1) judging by the interest from AI companies and venture capitalists, there is an enormous financial incentive to embed AI tools into all aspects of learning without robust evidence, (2) there is a general, nonevidence-based enthusiasm from some quarters about the impact of AI on learning, and (3) there is little evidence that unfettered access to AI actually does improve learning or cognitive (“critical thinking”) skills.

This does not mean that AI cannot support improving student engagement, learning outcomes, or course and instructor effectiveness, but rather, we have not yet figured out how to use it productively. Many reported uses of AI appear to have the potential (and are often used) to bypass learning,^19^ by providing a low barrier to finding answers that does not engage the cognitive mechanisms that are required for learning to occur.^20^ To facilitate actual learning, we need to consider what we already know about how people learn and build systems that take advantage of this knowledge. Currently, most students are left to their own devices and often default to asking chatbots for answers or asking the bots to complete assignments such as essays and reports.

There is no doubt about the potential of AI systems to improve learning outcomes. There are a several studies showing that use of a purposefully designed tutoring chatbot, one that provides hints and pointers but does not directly answer questions, can be more productive than nonpurposefully designed chatbots or than human tutors or than searching for answers on the Internet.^14,21^ The problem appears to be the unrestrained access to untrained AI chatbots that makes transfer of information from the bot to the completed task without passing through the student so easy. If learning were as simple as being told, every college lecture would be 100% effective, and we would not need universities (or schools). Here, using the admittedly limited research and evidence we currently have about the impact of AI on learning and the far larger research base for how learning occurs, I aim to provide a preliminary set of principles for the design of AI learning systems. These principles are intended for practitioners and researchers alike. They are undergirded by our current understanding of how learning occurs and how learning environments can be structured to support learning. They are not intended to be all-encompassing and should be expected to evolve in sophistication and practical value as more evidence is gathered.

## Overarching Principle: Learning Requires Effort and Engagement Which Can Easily Be Bypassed Using AI

Although most educators now recognize that “telling is not teaching”, in STEM disciplines, this has not significantly impacted the overall approaches to teaching and learning for many college instructors. The most prevalent approaches to transforming STEM teaching and learning in higher education have centered around student engagement strategies^22,23^ (often called active learning or evidence based instructional practices), that have been shown to improve outcomes for some students.^23^ There is also a smaller but growing literature that shows how carefully designed curricula and assessments can impact what students know, what they can do with that knowledge, and how they structure their disciplinary understanding.^24,25^ However, according to a national survey published in 2018 most STEM instruction is still based primarily on information delivery through lectures.^26^

One of the reasons for the low uptake of strategies intended to improve teaching and learning is almost certainly a lack of understanding about how people learn: learners must actively construct both their knowledge and how that knowledge can be used.^27,28^ Knowledge is not transmitted intact from instructor to learner but is reconstructed by the learner as they understand, connect, and use that knowledge. Thus, in a transmission-based learning environment (pure lectures), the actual learning typically takes place outside the class, when students study, do homework, or otherwise use the knowledge covered in class. Unfortunately, it is these activities that many students are replacing with AI support, for example, by asking for homework answers, brainstorming ideas, or writing essays, rather than wrestling with the work themselves.

Active learning techniques move the construction process into an environment where it can be guided by a knowledgeable instructor. When thoughtfully implemented, this approach has been found to be particularly helpful for students who have historically underperformed, but appears to be less important for those students who know how to actively engage without extra support.^23^ This latter group includes future college instructors and students whose background has provided them with the expertise and information to navigate the “hidden curriculum”.^29^ That is, college instructors typically were able to teach themselves, because of their background, intrinsic interest, motivation, and abilities. All of this may lead to a misunderstanding on the part of those same college instructors about why students are not learning from their excellent lectures.

Furthermore, as learners construct their knowledge base, many researchers believe that multiple cognitive and epistemic resources^30,31^ must be actively connected and integrated before students can develop a robust and useful cognitive framework. Unfortunately, there is no shortcut for the hard work of making sense of the world by productively connecting ideas, However, the use of AI chatbots is seductive; they are purposefully engineered to flatter and encourage interaction, and using them to support learning can be counterproductive as discussed earlier. Below I offer principles that align with what we know about learning intended to help instructors guide their students’ use of AI such that learning is accomplished. These principles are intended to support learning both disciplinary ideas and productive approaches to using those ideas; the intended audience is instructors, curriculum developers, and researchers who are interested in guiding students to use AI productively. They are not intended for the very different purpose of learning how to use AI systems for job and career preparation.

### Principle 1: Design (or Use) AI Learning Systems That Support Self-Regulated Learning

Successful learners are often characterized as self-regulated learners: they plan, monitor and evaluate their learning and learning strategies.^32–35^ However, students who use chatbots to provide whole cloth answers bypass any self-regulation or metacognitive strategies while offloading the business of knowledge construction and are, therefore, less likely to learn much. Since beginning learners are typically not in a position to evaluate responses, it is tempting for them to accept direct answers and move on without reflection. So it is not surprising that unsupervised chatbot use leads to a host of learning problems, including lack of retention of knowledge, less ownership of products produced, reductions in critical thinking and lower grades.^11–13,36^

Therefore, it is important to help students understand that while chatbots are readily available sources of information and homework answers, it is more effective to engage with the chatbot in a way that promotes an interactive and reflective partnership. However, a recent report from OpenAI “Building an AI-Ready Workforce” shows that the most common use of ChatGPT by college students is “Starting papers/projects”. That is, students most commonly begin their work on the chatbot, rather than making an initial effort and using the chatbot to refine, fill in gaps, or critique. The second most common use of ChatGPT was to “summarize texts”, and the third was “brainstorming”. All three (and many more user cases) imply that students are using GenAI in ways that circumvent rather than support learning. It is not surprising that transcribing (or copy-pasting) answers with minimal “edits”, leads to few memories of what was contained in the answer. We should not be surprised when the evidence tells us that using AI as the first step in any learning activity leads to less cognitive engagement, less recall of content, and lower abilities in summative assessments. Additionally brainstorming with AI has been shown to produce similar ideas, presumably because the AI has been trained on the same materials.^36^

However, learning is hard, and AI natives (present day middle and high schoolers and early college students) are inundated with social media posts that provide approaches to avoiding detection and in some cases encouraging cheating because “everyone does it”. It is easy to fall prey to the idea that letting the AI do the initial hard work of “thinking” will work out well. How then do we convince students to put in the work upfront and use AI to build on their own ideas and input, that is, to become self-regulated learners, rather than taking the easier way out and accepting the AI at face value? In a study of how students used AI to address a computational modeling task, Haraldsrud and Odden^37^ found that productive interactions with AI involved students using it as a “distributed cognitive system”, whereas when students outsourced their thinking to the AI, they often became overwhelmed and were unable to make sense of the output.

Designing AI systems that are intended to do more than provide answers to questions is certainly possible. There are now commercially designed tutoring systems which are becoming more available, and the big three AI companies (Open AI,^7^ Google Gemini,^8^ Claude Anthropic^38^) have released education versions of their systems for both students and instructors. ChatGPT, Claude, and Gemini all allow students to turn on “study mode” which will not provide answers but will prompt and lead students to appropriate answers. However, it is not at all clear how these systems are designed (what learning theories guide the bots design), nor do you know what information the system is calling upon to respond to the student query. Currently there is little research about what level of frustration students are willing to bear, nor how to stop them accessing a more accommodating bot that is friendly, engaging, and confident in their responses, but not necessarily correct, since GenAI systems can hallucinate and make up references. For a novice learner who almost certainly does not know enough, or has the experience-based confidence, to critique a chatbot output, passive acceptance of a ChatBot’s response can be seductive and seem completely reasonable. In addition, they can provide answers that are too complex, or too simple, or just biased: see MechaHitler,^39^ and may not emphasize or even address the intentions of the instructor, or engage the learner in reflection.

To address these problems, course-based Chatbot tutors using course materials and prompts engineered to the instructors’ specifications are relatively easy to develop. It is possible to load information (for example course materials) into the AI system, which can be used as sources of support and help for students. For example, instructors who are interested in helping students construct a causal mechanistic explanation for a phenomenon can load the module with examples and course materials that support students in this endeavor. There are also a number of resources available to help construct useful prompts.^7,8^ Examples include: a bot that provides hints and questions can engage a student in a reflective or Socratic conversation through which the student eventually arrives at an appropriate response, or a prompt that instructs the bot to be the learner, where the student teaches the bot. Even though this is our best theoretical approach, we still have little evidence about how students might interact with such a Socratic tutor “in the wild”. For example, a general chemistry study using a “think-pair-chatbot-share (TPCS)” approach showed promising results, compared to students engaging in think-pair-share (TPS), without support from a bot. However, participation was significantly lower in the TPCS condition.^40^ While it is much easier, and perhaps more initially satisfying to simply receive full feedback giving the correct information, evidence suggests that students often do not use feedback to improve.^41,42^

### Principle 2: Develop a Course Structure and Culture That Rewards the Journey (To Knowledge) Rather than the Correct Answer to Relatively Trivial Tasks

Many students see the work assigned to them as ″spending time”, and frankly in many cases, they are correct. For example, a lecture course where homework is assigned using a randomized multiple choice LMS, but is never mentioned again, might easily give students the impression that the homework is not actually necessary. In addition, AI systems excel at responding to such formulaic questions as well as to traditional tasks such as writing essays. We need to help students understand that the process of learning is important and design learning environments where moving straight to the product or answer is not the goal. That is, the reward is in the process of learning, not achieving the correct answer on a randomized multiple-choice test. This could take several forms, for example, by developing a course where both the act of learning and the result of learning are rewarded, or we can design activities that require evidence that the student initiated the first response to the task and followed up with how the student responded to feedback. For example, it is now possible not only to provide individual feedback to students using AI systems, but also to summarize the whole class responses for the instructor, who can then use that feedback to tailor their instruction.^43,44^ Other promising approaches in chemistry provide strategies for how instructors can adapt their courses to AI use, framing AI, modeling its use, providing AI compatible resources, and encouraging reflection and metacognition.^45,46^

An example of the possibilities for enhancing the focus on formative assessments is the curriculum “Chemistry, Life, the Universe and Everything, (CLUE)” which is specifically designed to engage students to use their knowledge of chemistry core ideas to construct scientifically plausible models, explanations, and arguments.^47^ Students practice these activities in both homework and group recitation sessions in which they are given credit for “good faith effort” rather than the correct response. They are encouraged to experiment, grapple with new ideas, and identify areas of confusion. Each of these activities can provide evidence of student activity, especially when delivered in a system that can capture student work in real time, such as beSocratic^48^ (an online formative assessment system that allows open ended, written, and drawn responses). The value of these formative assessment activities is shown by the fact that they contribute around 30–40% of the course grade, while course exams contribute around 60–65%. For those who may worry that the “rigor” of the course will be diminished by emphasizing formative tasks that focus on the process of learning, where students are rewarded for effort rather than correctness, the most pertinent evidence here is that this approach does not diminish the achievements of CLUE students. Even though more students pass the course when compared with students in a traditional course they score above average on an ACS conceptual general chemistry exam,^24^ and those students who move on to organic chemistry do just as well as other students from more selective courses (such as honors, majors, or self-selected learning communities).^49,50^ That is, students appear to benefit greatly from learning in such an environment and are more likely to pass the course and succeed in the next courses than students who were taught in a more traditional way that emphasized scores on summative assessments and correct answers to multiple choice homework problems.

Moving a significant proportion of the course expectations and grading to formative tasks, which can be designed to require student initial input, rather than relying solely on summative assessments and randomized homework systems (that can be answered by AI) is one approach to changing the learning environment that improves student outcomes and changes how students, instructors, and course designers think about learning. Using these student responses to drive learning in the next class can make the completion of these tasks more relevant and allow students to see where they fit in the learning process. However, the role of the summative exam should not be minimized at present. There must be a reliable way to determine what students have learned, but by emphasizing formative assessments as well, we can help students along that path to learning.

### Principle 3: Use AI as a Partner to Extend What Students Know and Can Do (Rather than to Reinforce Memorization and Algorithmic Problem Solving)

The availability of AI, which potentially has all the information available on the Internet, makes traditional assessment tasks (that emphasize memorization, rather than use of knowledge^51^) obsolete. This is not to say that there will be no need to learn facts and skills but rather that we can use the affordances of AI to go beyond these basics and learn them in the context of solving complex problems and designing solutions. Furthermore, this gives us an opportunity to consider what it is that we really want students to know and do by the end of the course. For example, instead of focusing on 263 content details described in the ACS General Chemistry Anchoring Concepts Map,^52^ the advent of AI could allow us to expand what we expect from students. If we focus on supporting students as they engage in scientific and engineering practices,^53^ or systems thinking,^54^ while using the content that they are learning from the AI system, we can not only develop more engaging tasks, but also provide better evidence about what students can do with their knowledge.

Part of addressing more complex problems is the act of brainstorming, that is, devising creative and innovative ideas for further exploration, and once again, students commonly believe that AI is beneficial for brainstorming.^55^ Initial evidence appeared to show that ideas generated by people using AI systems were in fact more creative than any one person without the aid of AI.^56^ However, a subsequent study showed when the ideas from groups of people are combined the overall set of ideas is more creative and more diverse than for groups using AI.^36^ As the authors note “reliance on ChatGPT for idea generation comes with a trade-off: while enhancing the creativity of individuals, it reduces the diversity of ideas in a pool of ideas, a critical element for effective brainstorming.” It is not surprising that individuals using the same AI bots for brainstorming will produce highly similar sets of ideas. That being said, it has been shown that teams of people working with AI as a partner (rather than an initiator or deliverer of information) were more productive and innovative than teams working alone, or individuals working with AI.^57^ Therefore, it seems likely that using AI as a partner for teams of students acting as a resource as they design solutions to problems shows great potential. Similarly it is highly likely that AI can be used to support learning environments that are based on Explore–Invent–Apply learning approaches, such as POGIL.^58^

This kind of problem^59^ or project based^60^ learning has a long and storied history most often in precollege or medical school environments, but in chemistry education this approach has not been widely adopted outside of laboratories,^61,62^ perhaps because of the difficulty of enacting it with large classes. However, it now seems entirely possible that by using AI as a learning partner students can be asked to engage with far more complex problems than are typical at present, by asking students to explore relevant phenomena that involve not only understanding and using the underlying chemistry, but also real-world constraints such as broader ecological, societal, and political systems.^63^ For example, the problem of polyfluorinated alkyl substances (PFAS) in the environment, can serve as the basis for learning a wide range of chemistry principles (intermolecular forces, bond energies, solubility, reactivity) while at the same time engaging students in designing and evaluations solutions to problems.^64^ At the same time, AI bots can help the instructor design and align such instructional tasks with appropriate prompting.^43^

### Principle 4: AI Use Should Be Equitable and Clearly Delineated

While many students report using AI in their educational endeavors, in fact there is a significant group of students who do not.^65^ There are several reasons for this, including the recognition that offloading cognition is not conducive to learning, concerns about appropriate use, lack of access to AI systems, and lack of knowledge. Because most instructors and institutions are still in a state of flux, the rules of engagement with AI have often not been made clear to students. Couple this with genuine confusion about what constitutes cheating, and we have a situation that is both unfair and inequitable. There will always be some students who use AI to complete assignments, even if an instructor has decided that absolutely no use of AI is permissible. In such a situation, AI use may allow students to complete work (often without learning much as discussed earlier) and be given a good grade, while at the same time a student who completes assignments in good faith may make errors and receive a lower grade. To address this unfairness, if no AI is allowed, then no work outside of class time should be counted as part of a grade. Only summative in-person examinations or tasks, where there is no access to AI, should be acceptable in this situation; admission tests (MCAT, LSAT) and certification exams are run in this way.

Certainly, retreating to the precomputer age is one approach to handling the impact of AI, and it would ensure fairness of a sort since no-one could use outside help. However, there is an argument that ignoring technologies such as chatbots is short-sighted, since we are not able to put the genie back in the bottle. Additionally, there is ample evidence that relying solely on summative assessments tends to favor particular groups of students. It has been shown that grades in a typical general chemistry course, where numerical problem solving and rote skills are emphasized, are highly correlated with math preparation.^66^ In contrast, courses that focus on the use of scientific practices, with opportunities to engage in formative tasks, are less likely to show such correlations–that is they are more equitable.^24^

The alternative is to take advantage of the advantages of AI as outlined in these principles. Students deserve to know how and why they should use AI to support learning, and they should also have access to such systems and be taught how to use them effectively.

## Conclusions

This paper is intended to provide guidance for instructors, researchers, and curriculum developers about how to incorporate artificial intelligence teaching and learning strategies into their practice. I propose that we do need to consider changes in the design of learning environments, how we teach, and what we expect students to know and to do. Recognizing that students must actively engage in their learning is crucial; learning something new is difficult, and while AI may provide ways to support students through that difficulty, it cannot replace student engagement. Therefore, we need to ensure that (1) students are supported in their learning by encouraging interaction, reflection, and construction of new ideas and skills; (2) we recognize the journey to learning not just the end point, by providing scaffolding and formative tasks that are recognized and rewarded in the overall course structure; (3) we use AI to extend what has been possible, particularly in large enrollment courses, rather than emphasize the rote and skills based competencies that are often the end point of such courses; and (4) we ensure that we treat students fairly and equitably with regard to AI access.

Business as usual will result in a generation of students who are unlikely to develop the cognitive skills that will be required in the future. If that were not enough, consider the fate of instructors who do not adapt, who continue to emphasize facts and skills that can be developed by AI tutoring systems rather than reconsidering what it is that is important for students to know and do. Their fate (at best) will be that of what has come to be known as the reverse centaur,^67^ that is “someone who is harnessed to the machine, reduced to a mere peripheral for a cruelly tireless robotic overlord that directs you to do the work that it can’t”.^67^ While this may seem dramatic, it may well be that failing to adapt will lead to a new class of instructor. One who is perhaps in charge of thousands of students, who are interacting with bots and who are assessed by bots. The instructor will be there to ensure that the bots are functioning and to adjudicate errors. This is clearly the vision of some tech leaders who have a strong financial stake in that future. To avoid this, we need to act now, to determine how AI can help students learn, not just in the old ways, but to prepare them for the future. It is time to seriously reconsider what is important for students to know and do, and what does it mean to learn in the age of AI. Personally, I would rather be the centaur “someone whose work is supercharged by automation: you are a human head atop the tireless body of a machine that lets you get more done than you could ever do on your own.” Now is the time to take control of the reins, to redesign learning systems that can take advantage of the affordances of AI, without outsourcing the creative and rewarding parts of teaching and learning.
